# Supplementary material for: Mobility and non-household environments: Understanding dengue transmission patterns in urban contexts
Source: PLoS Negl Trop Dis. 2026 Jul 2;20(7):e0014487. doi: 10.1371/journal.pntd.0014487 (PMC13354100; doi:10.1371/journal.pntd.0014487)
Supplement: S1 Text — (PDF) [file pntd.0014487.s001.pdf]

Supporting Text

Mobility and non-household environments: understanding dengue transmission patterns in urban contexts

Víctor Hugo Peña-García[1, *], Bryson A. Ndenga[2], Francis M. Mutuku[3], Donal Bisanzio[4], A. Desiree LaBeaud[5], Erin A. Mordecai[1]

[1] Department of Biology, Stanford University, Stanford, CA, USA

[2] Kenya Medical Research Institute, Kisumu, Kenya

[3] Department of Environmental and Health Sciences, Technical University of Mombasa, Mombasa, Kenya

[4] Department of Veterinary Sciences, University of Turin, Turin, Italy

[5] Department of Pediatrics, Division of Infectious Disease, School of Medicine, Stanford University, Stanford, CA, USA

Supporting Methods

Mosquito movement parameterization

We consider two variables to estimate a baseline migration probability for the number of mosquitoes moving from given locations: availability of both breeding and blood-feeding resources.

The model includes a local density-dependent function that allows for the mosquito population to grow in a location-specific way, allowing it to represent dynamics previously described for a fragmented environment [1]. By using this function, the number of mosquitoes in each location grows according to availability of water containers, the density of immature mosquitoes in those containers, and temperature (peaking at 29°C), as follows:

$$f(D) = \frac{1}{1 + e^{0.09D - 0.55}} d(T) \quad (1)$$

Where

$$d(T) = -0.166 + 0.08T - 0.0014T^2 \quad (2)$$

In the equation, D is the larval density expressed as the ratio of the number of larvae to liters of water available for breeding in the structure, and $d(T)$ is the term describing the temperature-dependence of population growth. The equation allows the sub-population to grow when larval density is low by assuming they occupy all the water containers inside or around the structure. As the mosquito sub-population grows, resource availability in the water declines, slowing population growth. By considering Nm_t as the mosquito subpopulation size at time t, when the ratio Nm_t/Nm_{t+1} approaches 1, breeding site resources are reaching their carrying capacity and hence, the probability of a mosquito migrating increases. Because there is a strong stochastic component in estimating Nm_{t+1}, by running 20 simulations for every location every day, we estimated a parameter MP as the median of ratios Nm_t/Nm_{t+1}.

We also consider the probability that each female mosquito bites a human within a given location. The rate of biting is temperature dependent (a), according to previously published work [2]. We coupled it into a binomial distribution to define the number of biting mosquitoes as follows:

$$Nb \sim Bin(Nm_t, a) \quad (3)$$

Where Nb is the number of females needing to bite on day t and Nm_t is the size of sub-population mosquitoes that day. While most models assume that all females bite when they

need to, here we consider the possibility that mosquitoes cannot find blood meal hosts in a given place and time as dependent on the human occupancy of a given location. We assume that there is a probability for Nb mosquitoes to have a successful biting human-mosquito encounter ($P(bite)$) based on the size of Nb and the amount of time that humans spend in the location, as follows:

$$P(bite) = 1 - e^{-\frac{h}{24}Nh_t} \tag{4}$$

Where Nh_t is the number of people attending a given location on day t for a structure-specific number of hours. Finally, the actual number females that fed (NF) in each day, is estimated by

$$NF \sim Bin\big(Nb, P(bite)\big) \tag{5}$$

In this sense, when the number of people on a given day is low and the number of hours they spend in the location are few, the probability of a female mosquito successfully biting is low. So, as the ratio NF/Nb (later called MF) approaches 0, the probability of mosquitoes migrating increases. Again, for every location every day, 20 simulations were run to estimate a distribution of MF.

Finally, we estimated the migration probability as the product $Mp\cdot(1\text{-}MF)$, which depends on both larval density conditions via Mp and adult biting conditions via MF. The resulting probability was used in a binomial distribution to determine the number of migrating mosquitoes each day. To evaluate the effect of different mosquito movement regimes on the burden of dengue, we considered migration rates of 100% (as unmodified product $Mp\cdot(1\text{-}MF)$), 50%, and 10%. The total number of cases was quantified after 200 simulations.

Once a mosquito migrates, a new location is assigned by considering a dispersal kernel [3]. Following previous work [4], we used a lognormal function with the form

$$PD = \frac{1}{(2\pi)^{\frac{3}{2}}bd^2} e^{-\frac{log\left(\frac{d}{a}\right)^2}{2b^2}} \tag{6}$$

Where d is distance and both a and b are parameters to be estimated. We fitted a function by assuming a mean dispersal distance of 105.69 meters, as estimated for *Aedes aegypti* by [5].

Use of temperature-dependent function into the model

Biological and transmission-related traits of mosquitoes are strongly relying on temperature. The mathematical formulations and their implementation within the model are described in S1 Table as reported previously [6].

Supporting results

Movement of mosquitoes is costly and reduces human dengue burden

When we considered different regimes of mosquito movement, the model yielded a lower number of cases when mosquito migration was higher. The median number of cases was 306 (IQR: 222 – 419) at 100% of mosquito movement and increased to 1,090 (IQR: 533 – 1,714) and 4,670 (IQR: 4,005 – 5,229) when number of mosquitoes was 50% and 10%, respectively (S4 Table). Still the proportion of infections happening in HH compared to NH remained lower regardless of mosquito movement (Figures S8). To better understand the mechanism by which mosquito movement was reducing transmission, we studied the size of mosquito populations for each mosquito movement regime, which resulted in smaller population sizes at higher movement rate (Figure S9), indicating the importance of mosquito mortality during migration as a limit on transmission.

Supporting Discussion

In this model we also included estimates of mosquito movement, where we found that high levels of mosquito migration induce high mosquito mortality, thereby reducing mosquito abundance and transmission (Figures S8 and S9). These results suggest that mosquito migration is carried out at a high cost for the individual and population, which supports the empirical observation that mosquitoes tend to stay in or close to the same location where they are breeding. Though our model has different parameterization and purpose from that previously reported by Reiner and colleagues, they also included both movement of people and mosquitoes and found that the former is the real force shaping dengue transmission as opposed to movement of mosquitoes, which diffuses it [7]. Interestingly, they found that their model output matches real observations when mosquito movement is decreased and, under certain scenarios, when mosquito movement equals to zero [7].

In our model, a migrant mosquito travels to another location, irrespective of the suitability of that location (i.e., presence of water containers or established mosquito population), with a distance-based probability. If the new location is suitable for breeding, a new mosquito subpopulation is established, otherwise the migrant mosquito will die before reproducing. A migrant mosquito may be more attracted to travel to a location with water containers, as

suggested by other studies [8], which is not included in the model. However, it is not known how likely it is for a migrating mosquito to travel longer distances in search of containers during migration if the closest location does not have any. This type of process has never been studied before. Another variable not considered is the number of buildings, roads, and other urban features that might impose some limits to mosquito dispersal like those described previously, where all *Ae. aegypti* individuals were recaptured in the same block where they were released, unable to cross roads [9].

Though there are several mark-release-recapture studies recording long travel distances by *Ae. aegypti*,[5] most of these studies are artificially releasing mosquitoes where they are forced to travel looking for a place to settle. While these studies are useful to evaluate the capacity of mosquitoes to fly in these conditions, they are not indicative of the propensity for already settled mosquitoes to travel the distances recorded in these studies.

According to results of this model, intra-urban dispersion of mosquitoes is not explained by mosquito migration. A plausible explanation is that dispersion is actively driven by human mobility. This would underscore the importance of human movement by suggesting that it is mainly responsible for the burden and dispersal of dengue through communities. Though the role of human mobility on intra-urban mosquito dispersion is hard to measure, there are several studies explaining the importance of human movement in dengue transmission at different space levels [7,10-15].

References

1. McCormack, C.P.; Ghani, A.C.; Ferguson, N.M. Fine-scale modelling finds that breeding site fragmentation can reduce mosquito population persistence. *Commun Biol* **2019**, *2*, 273, doi:10.1038/s42003-019-0525-0.

2. Mordecai, E.A.; Cohen, J.M.; Evans, M.V.; Gudapati, P.; Johnson, L.R.; Lippi, C.A.; Miazgowicz, K.; Murdock, C.C.; Rohr, J.R.; Ryan, S.J.; et al. Detecting the impact of temperature on transmission of Zika, dengue, and chikungunya using mechanistic models. *PLoS Negl Trop Dis* **2017**, *11*, e0005568, doi:10.1371/journal.pntd.0005568.

3. Nathan, R.; Klein, E.; Robledo-Arnuncio, J.J.; Revilla, E. Dispersal kernels: review. In *Dispersal Ecology and Evolution*, Clobert, J., Baguette, M., Benton, T.G., Bullock, J.M., Eds.; Oxford University Press: 2012; p. 0.

4. Marcantonio, M.; Reyes, T.; Barker, C.M. Quantifying *Aedes aegypti* dispersal in space and time: a modeling approach. *Ecosphere* **2019**, *10*, e02977, doi:https://doi.org/10.1002/ecs2.2977.

5. Moore, T.C.; Brown, H.E. Estimating *Aedes aegypti* (Diptera: Culicidae) Flight Distance: Meta-Data Analysis. *J Med Entomol* **2022**, *59*, 1164-1170, doi:10.1093/jme/tjac070.

6. Peña-García, V.H.; LaBeaud, A.D.; Ndenga, B.A.; Mutuku, F.M.; Bisanzio, D.; Andrews, J.R.; Mordecai, E.A. Non-household environments make a major contribution to dengue transmission: implications for vector control. *R Soc Open Sci* **2025**, *12*, 241919, doi:10.1098/rsos.241919.

7. Reiner, R.C.; Stoddard, S.T.; Scott, T.W. Socially structured human movement shapes dengue transmission despite the diffusive effect of mosquito dispersal. *Epidemics* **2014**, *6*, 30-36, doi:10.1016/j.epidem.2013.12.003.

8. Richter, O.; Nguyen, A.; Nguyen, T. Application of reaction-diffusion equations for modeling human and breeding site attraction movement behavior of *Aedes aegypti* mosquito. *Math Biosci Eng* **2022**, *19*, 12915-12935, doi:10.3934/mbe.2022603.

9. Trewin, B.J.; Pagendam, D.E.; Zalucki, M.P.; Darbro, J.M.; Devine, G.J.; Jansen, C.C.; Schellhorn, N.A. Urban Landscape Features Influence the Movement and Distribution of the Australian Container-Inhabiting Mosquito Vectors *Aedes aegypti* (Diptera: Culicidae) and *Aedes notoscriptus* (Diptera: Culicidae). *J Med Entomol* **2020**, *57*, 443-453, doi:10.1093/jme/tjz187.

10. Stoddard, S.T.; Morrison, A.C.; Vazquez-Prokopec, G.M.; Paz Soldan, V.; Kochel, T.J.; Kitron, U.; Elder, J.P.; Scott, T.W. The role of human movement in the transmission of vector-borne pathogens. *PLoS Negl Trop Dis* **2009**, *3*, e481, doi:10.1371/journal.pntd.0000481.

11. Stoddard, S.T.; Forshey, B.M.; Morrison, A.C.; Paz-Soldan, V.A.; Vazquez-Prokopec, G.M.; Astete, H.; Reiner, R.C.; Vilcarromero, S.; Elder, J.P.; Halsey, E.S.; et al. House-to-house human movement drives dengue virus transmission. *Proc Natl Acad Sci U S A* **2013**, *110*, 994-999, doi:10.1073/pnas.1213349110.

12. Phaijoo, G.R.; Gurung, D.B. Modeling Impact of Temperature and Human Movement on the Persistence of Dengue Disease. *Comput Math Methods Med* **2017**, *2017*, 1747134, doi:10.1155/2017/1747134.

13. Tocto-Erazo, M.R.; Olmos-Liceaga, D.; Montoya-Laos, J.A. Effect of daily human movement on some characteristics of dengue dynamics. *Math Biosci* **2021**, *332*, 108531, doi:10.1016/j.mbs.2020.108531.

14. Ospina-Aguirre, C.; Soriano-Paños, D.; Olivar-Tost, G.; Galindo-González, C.C.; Gómez-Gardeñes, J.; Osorio, G. Effects of human mobility on the spread of Dengue in the region of Caldas, Colombia. *PLoS Negl Trop Dis* **2023**, *17*, e0011087, doi:10.1371/journal.pntd.0011087.

15. Bomfim, R.; Pei, S.; Shaman, J.; Yamana, T.; Makse, H.A.; Andrade, J.S.; Lima Neto, A.S.; Furtado, V. Predicting dengue outbreaks at neighbourhood level using human mobility in urban areas. *J R Soc Interface* **2020**, *17*, 20200691, doi:10.1098/rsif.2020.0691.
