# Supplementary figures and images for: Mobility and non-household environments: Understanding dengue transmission patterns in urban contexts
Source: PLoS Negl Trop Dis. 2026 Jul 2;20(7):e0014487. doi: 10.1371/journal.pntd.0014487 (PMC13354100; doi:10.1371/journal.pntd.0014487)

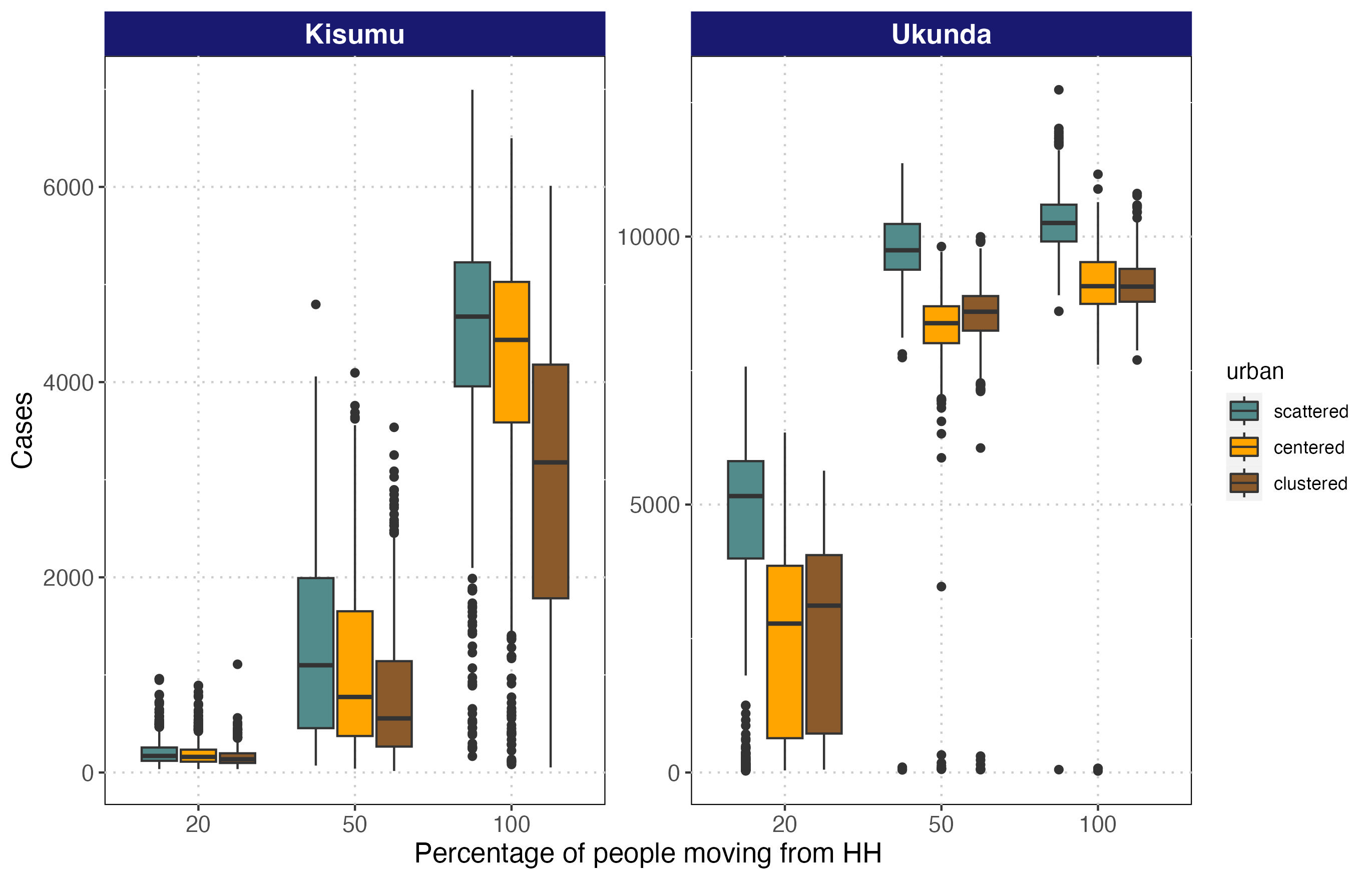

Supplement: S1 Fig — A higher burden of dengue at scattered distribution of NH is also evident for both Kenyan cities of Kisumu and Ukunda. The horizontal line indicates the median of 200 runs, while box represents interquartile range (IQR). Whiskers show the range of 1.5·IQR extending beyond the box. Dots are data points outside the whole range. (JPEG) [file pntd.0014487.s002.jpeg]

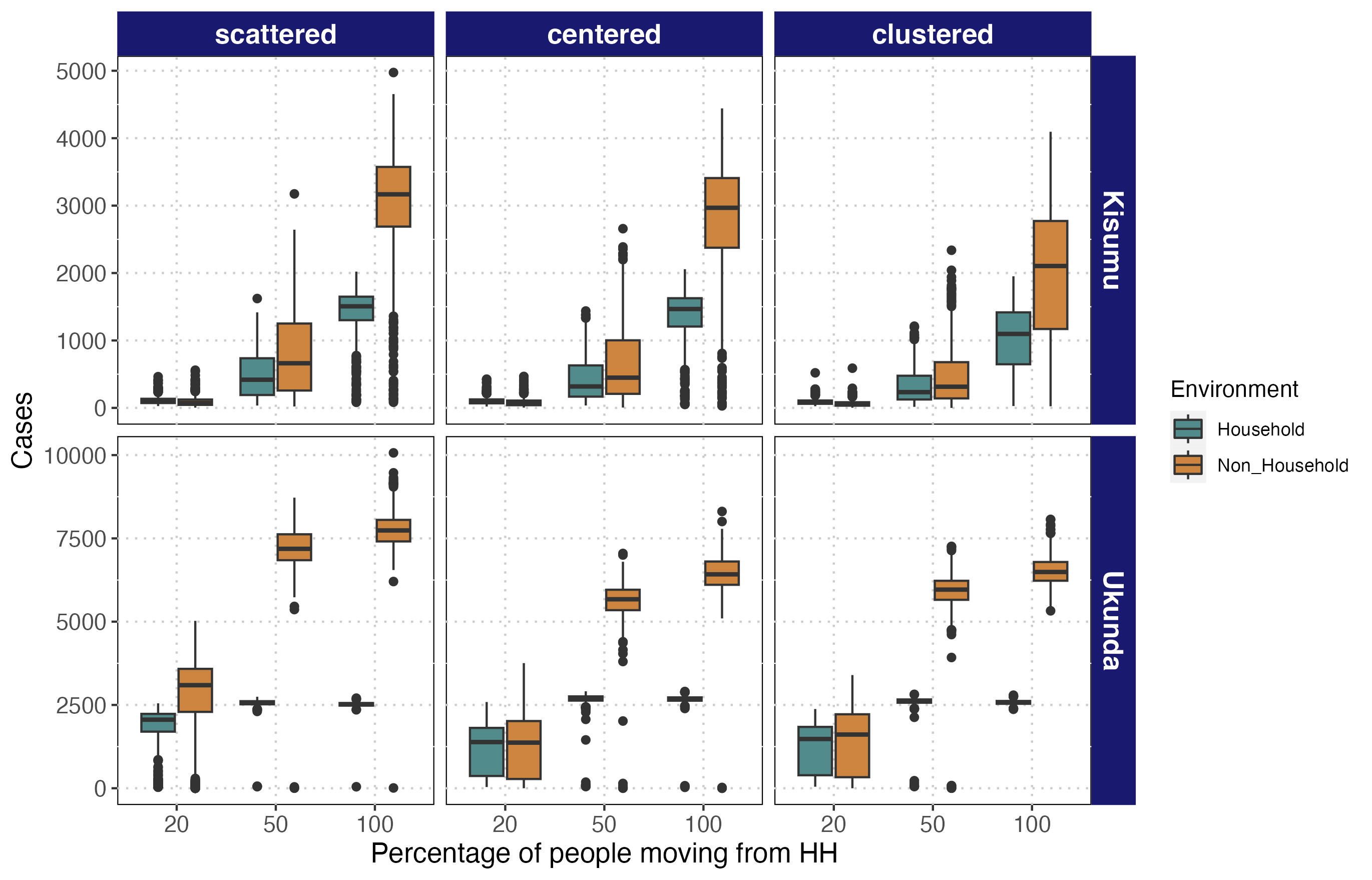

Supplement: S2 Fig — The boxes show the IQR of cases of 200 runs for each simulation, median is represented by horizontal line, whiskers represent the range beyond the box of 1.5·IQR, and the dots are data points outside such range. (JPEG) [file pntd.0014487.s003.jpeg]

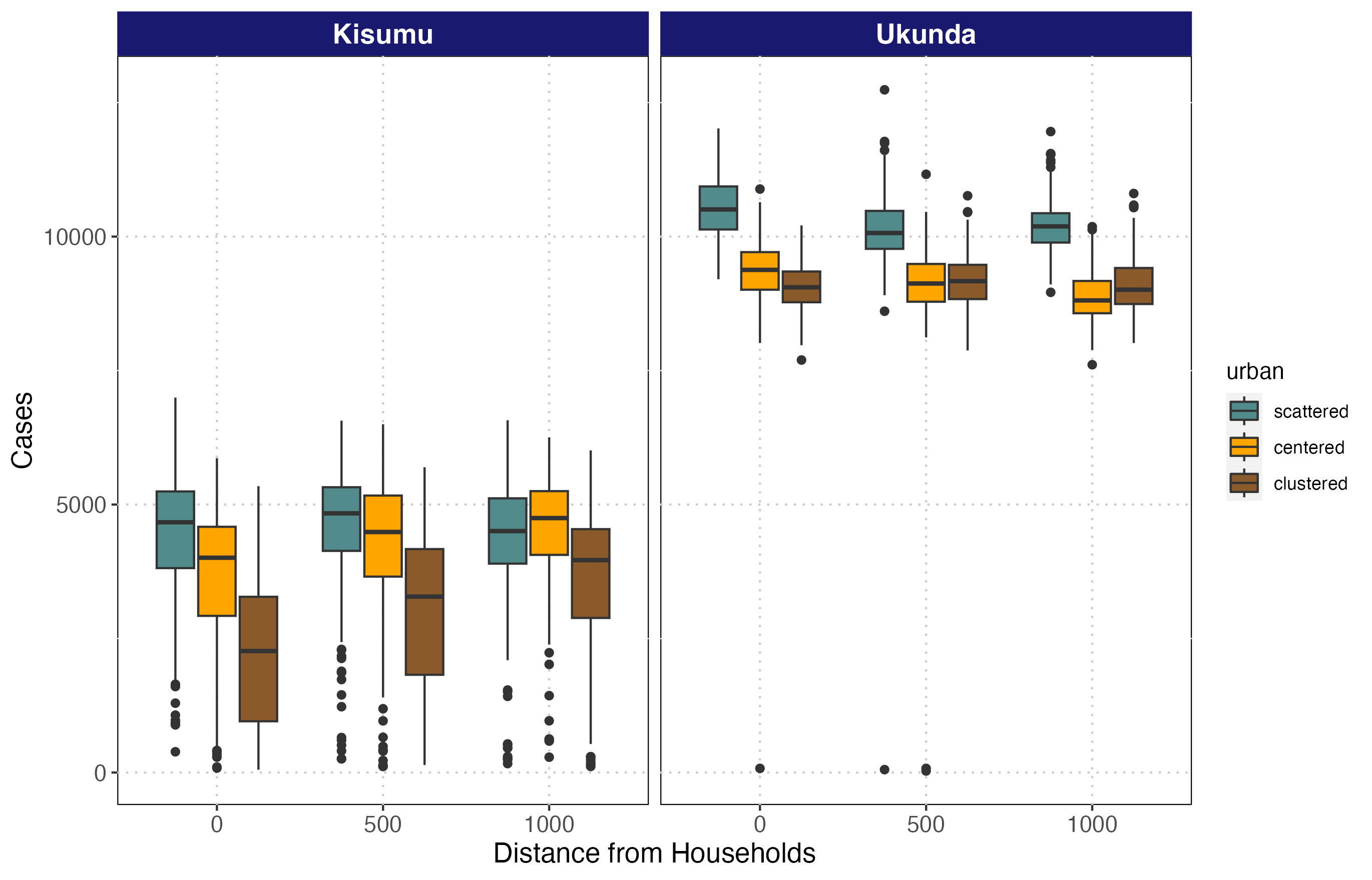

Supplement: S3 Fig — A higher burden is achieved when NH are randomly spatially distributed (scattered). Three levels of distance were assessed (the closest NH from HH [categorized as 0], at least 500 meters, and at least 1000 meters). (JPEG) [file pntd.0014487.s004.jpeg]

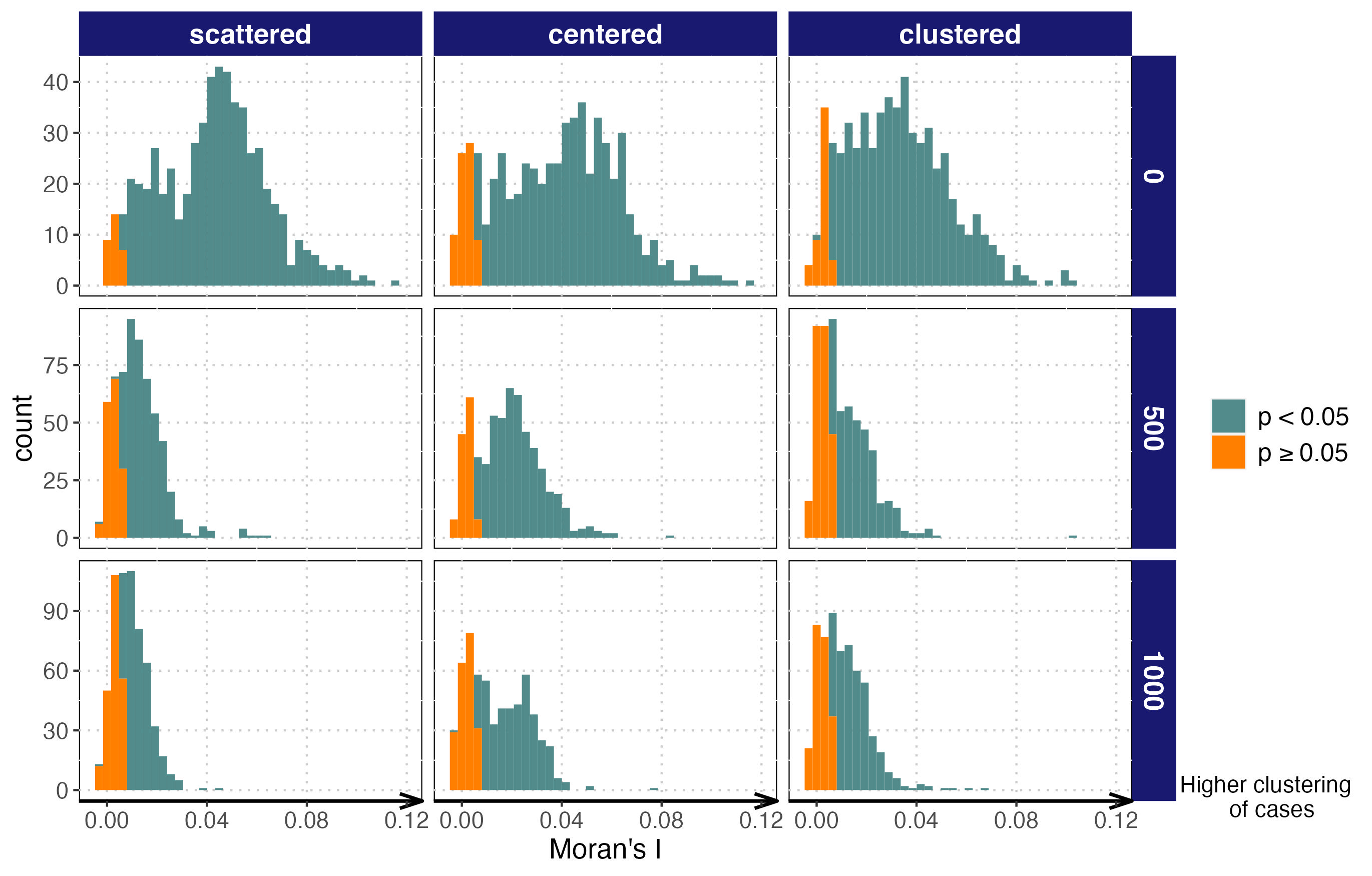

Supplement: S4 Fig — Distribution of values are shown across three different urban conformations and different distance regimes (distance traveled by people from HH to NH: the closest distance [categorized as zero], and at least 500 and 1000 meters). Significance with α = 0.05 is shown according to color. The level of clustering of dengue cases increases with Moran’s I value. (JPEG) [file pntd.0014487.s005.jpeg]

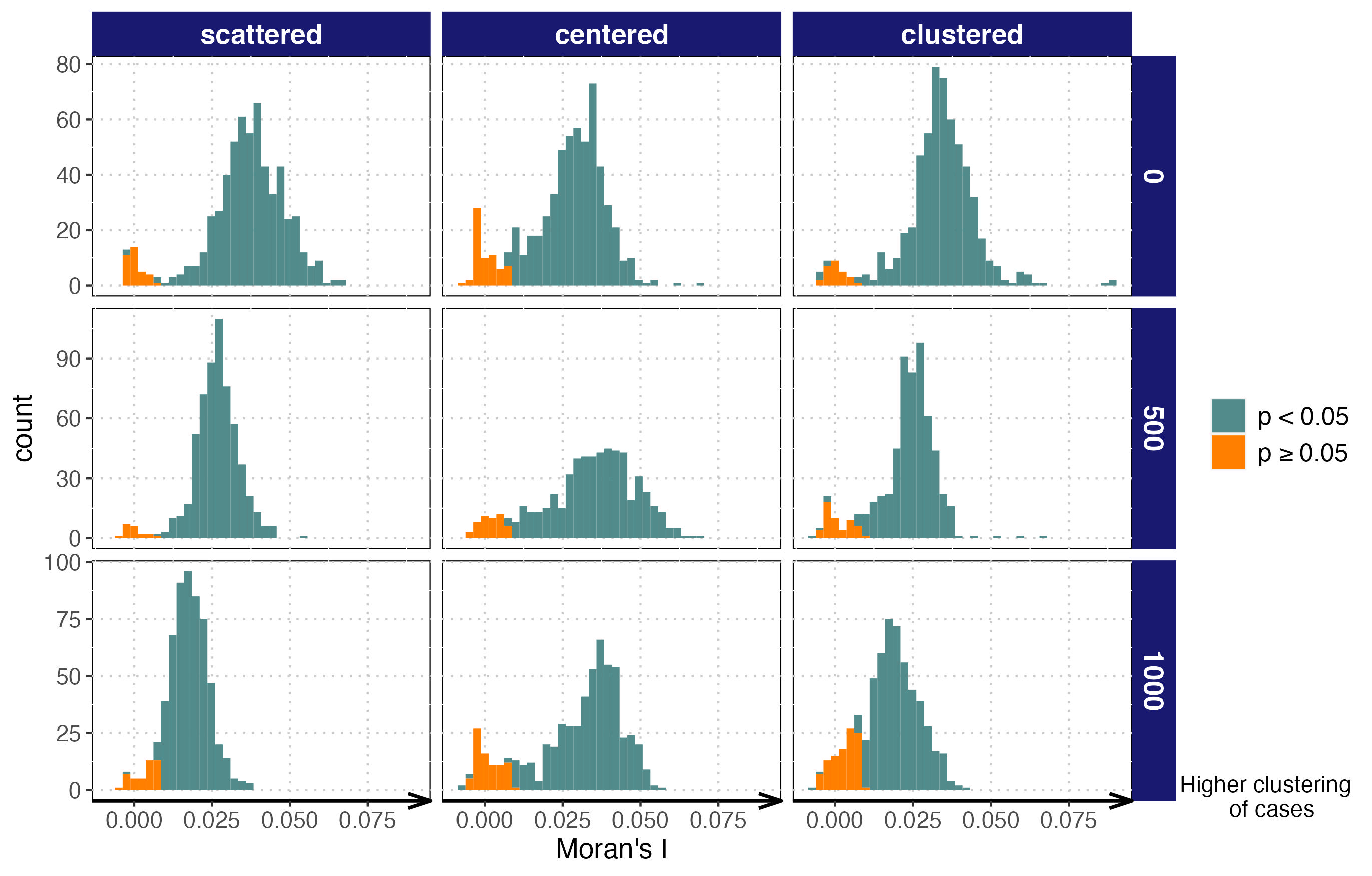

Supplement: S5 Fig — Distribution of values are shown across three different urban conformations and different distance regimes (distance traveled by people from HH to NH: the closest distance [categorized as zero], and at least 500 and 1000 meters). Significance with α = 0.05 is shown according to color. The level of clustering of dengue cases increases with Moran’s I value. (JPEG) [file pntd.0014487.s006.jpeg]

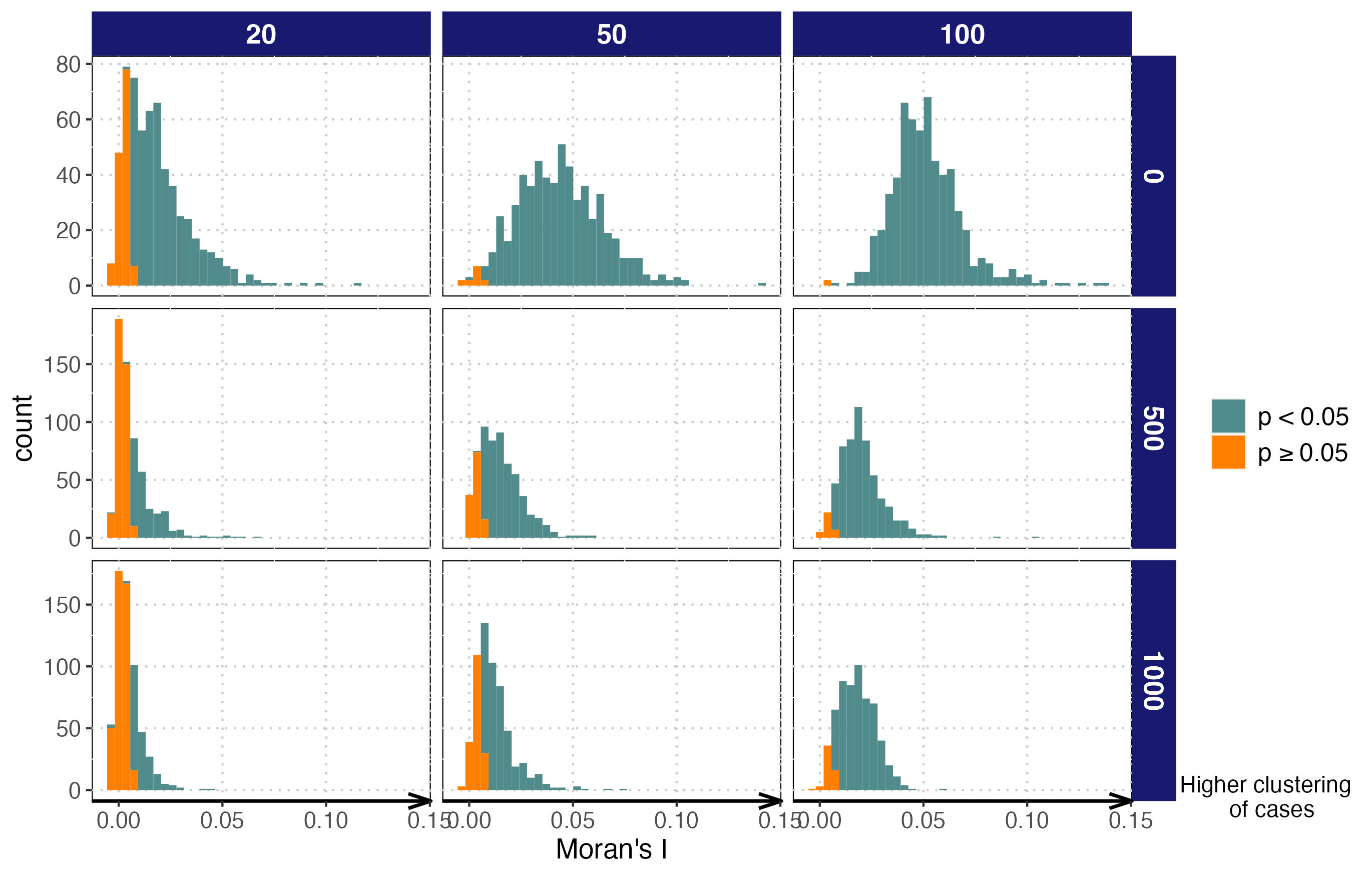

Supplement: S6 Fig — Distribution of values are shown across three different percentages of people travelling and different distance regimes (distance traveled by people from HH to NH: the closest distance [categorized as zero], and at least 500 and 1000 meters). Significance with α = 0.05 is shown according to color. The level of clustering of dengue cases increases with Moran’s I value. (JPEG) [file pntd.0014487.s007.jpeg]

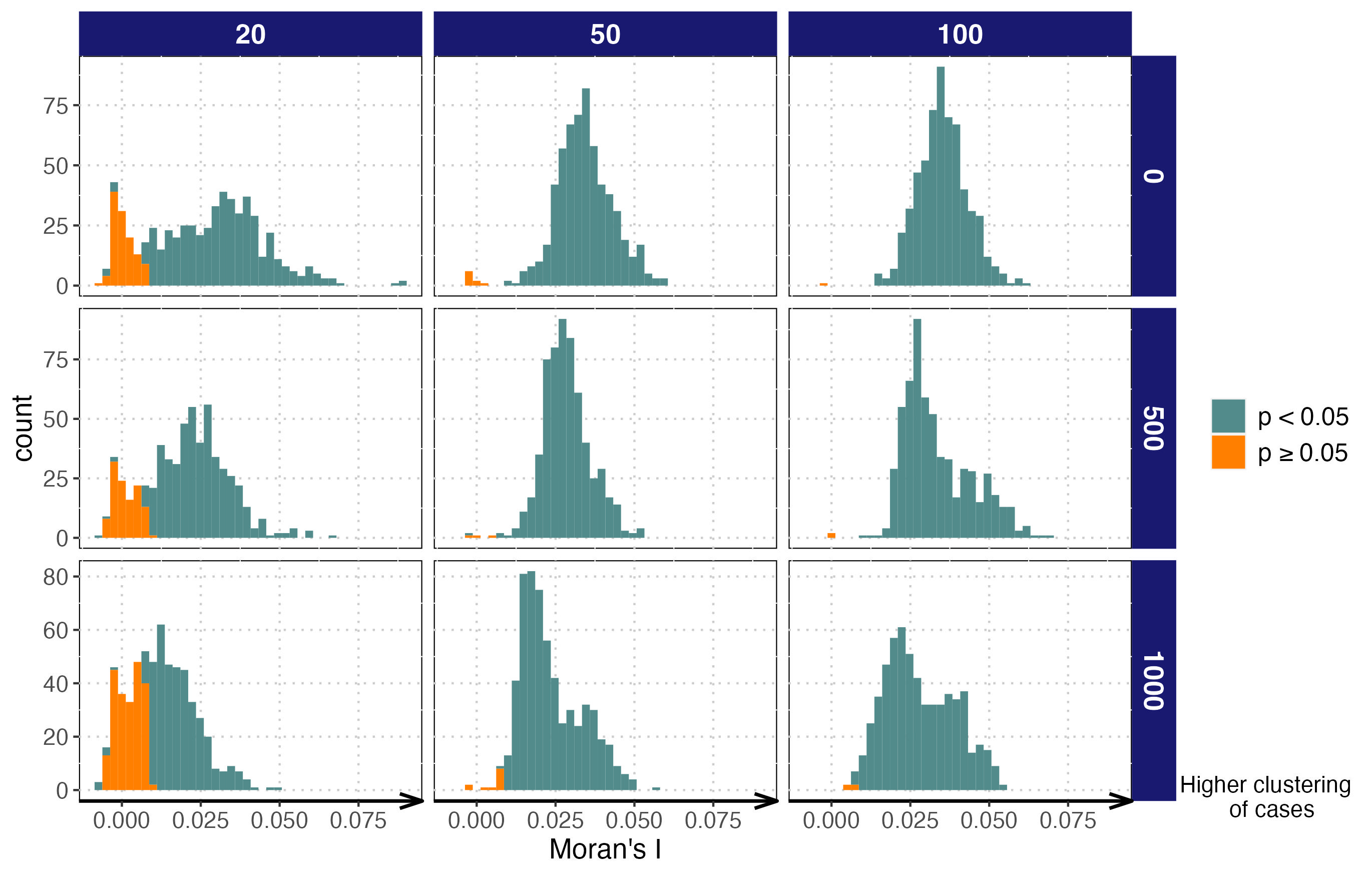

Supplement: S7 Fig — Distribution of values are shown across three different percentages of people travelling and different distance regimes (distance traveled by people from HH to NH: the closest distance [categorized as zero], and at least 500 and 1000 meters). Significance with α = 0.05 is shown according to color. The level of clustering of dengue cases increases with Moran’s I value. (JPEG) [file pntd.0014487.s008.jpeg]

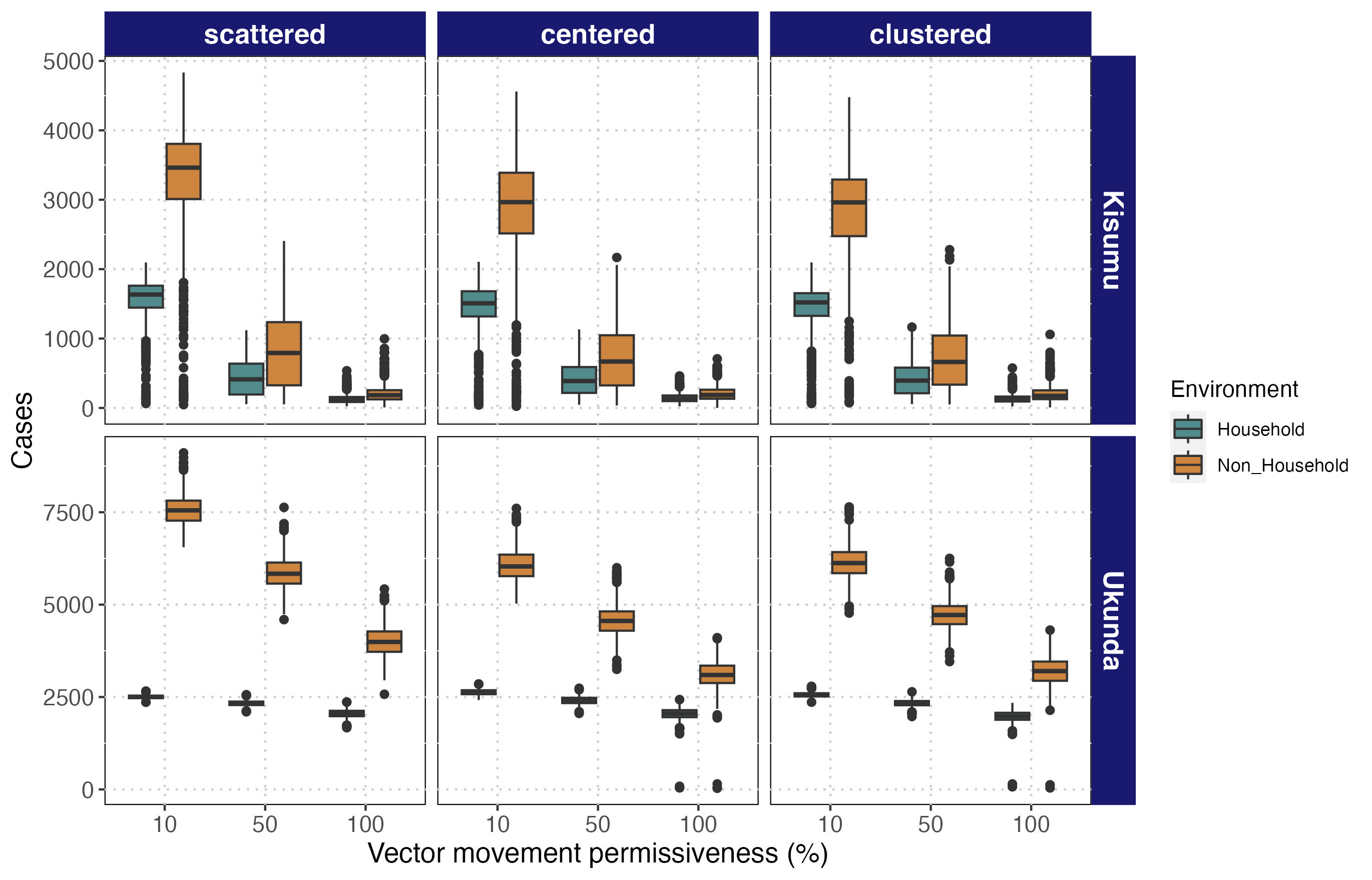

Supplement: S8 Fig — Difference on the number of infections happening in HH and NH and in turn the burden of dengue decreases as mosquito movement decreases as well. Boxplots are showing the distribution of the number of cases for three levels of mosquito movement (x axis) and three urban conformations (top panels) for two Kenyan cities (Kisumu and Ukunda). (JPEG) [file pntd.0014487.s009.jpeg]

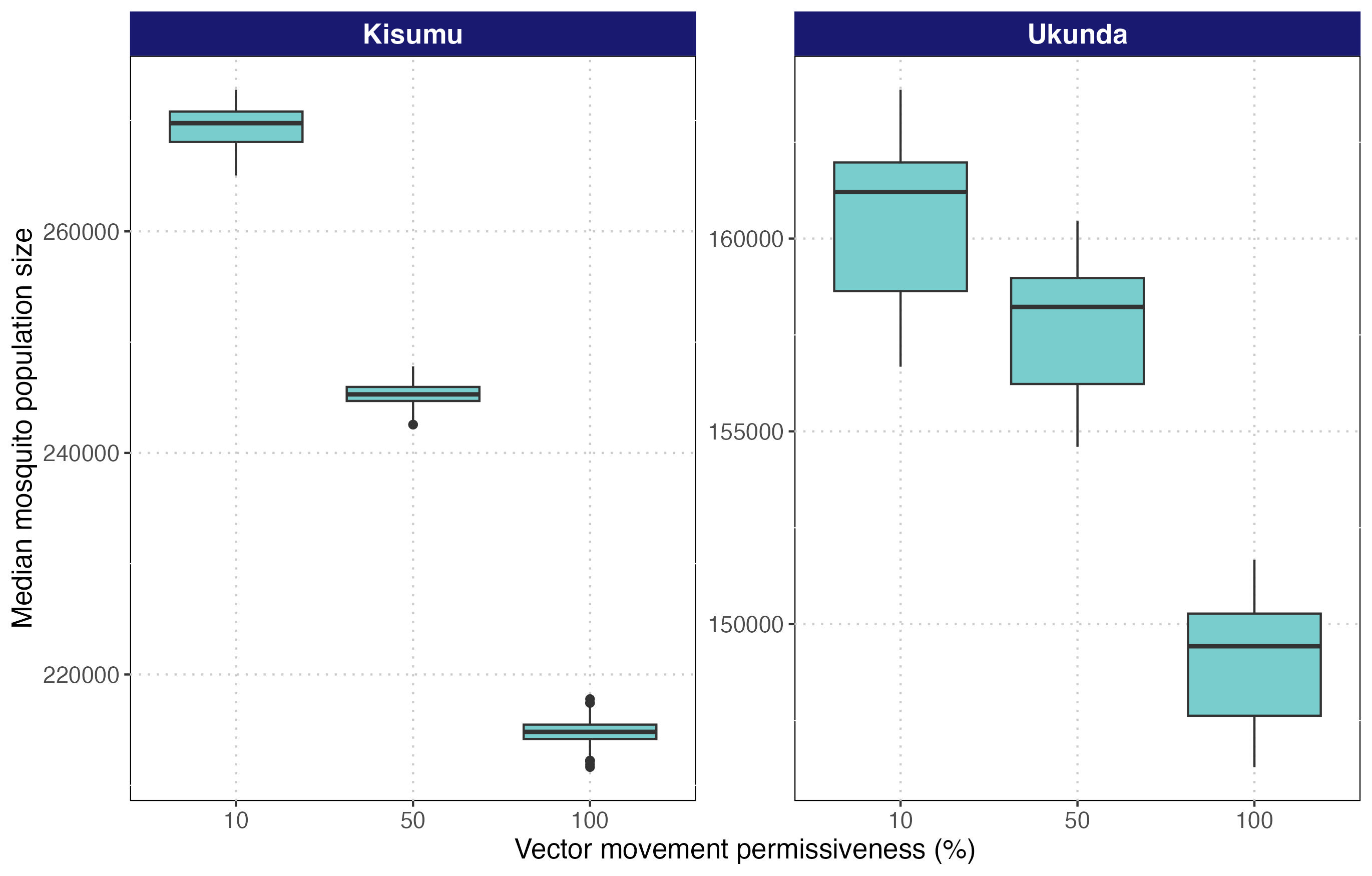

Supplement: S9 Fig — Boxplots show the distribution of the median size of population size across the two modelled years for 200 runs for two Kenyan cities (Kisumu and Ukunda). (JPEG) [file pntd.0014487.s010.jpeg]
