# Supplementary material for: Mobility and non-household environments: Understanding dengue transmission patterns in urban contexts
Source: PLoS Negl Trop Dis. 2026 Jul 2;20(7):e0014487. doi: 10.1371/journal.pntd.0014487 (PMC13354100; doi:10.1371/journal.pntd.0014487)
Supplement: S1 Table — Functions were extracted from previous works originally fitted by Mordecai [2] and later modified and used by Huber [7] and Caldwell [8]. Functions can be either Brière [cT(T-Tmin)(Tmax-T)1/2] or quadratic [c(T-Tmax)(T-Tmin)]. (DOCX) [file pntd.0014487.s011.docx]

**S1 Table:** **Implementation of mosquito-related functions to model population dynamics traits.** Functions were extracted from previous works originally fitted by Mordecai [2] and later modified and used by Huber [1] and Caldwell [2]. Functions can be either Brière [*cT(T-T_min_)(T_max_-T)^1/2^*] or quadratic [*c(T-T_max_)(T-T_min_)*].

| **Trait** | **Estimation function** | | | | **Use** |
| --- | --- | --- | --- | --- | --- |
|  | **function** | **c** | **Tmin** | **Tmax** |  |
| Man biting rate (*a*) | Brière | 2.02e^-04^ | 13.35 | 40.08 | $N_{B}\sim Bin\left( Nm,a \right)$* |
| Mortality rate (*µ*) | Quadratic | -1.48e^-01^ | 9.16 | 37.73 | *N_D_*~𝐵𝑖𝑛(𝑁𝑚, 𝜇)^+^ |
| Probaility of infection of a mosquito (*c*) | Brière | 4.91e^-04^ | 12.22 | 37.46 | $vc=b\cdot c$^†^  𝐸~𝐵𝑖𝑛(*N*_𝑏𝑖𝑡_,𝑣𝑐)^‡^ |
| Probaility for a mosquito to become infectious (*b*) | Brière | 8.49e^-04^ | 17.05 | 35.83 |  |
| Parasite development rate (*PDR*) | Brière | 6.65e^-05^ | 10.68 | 45.90 | $inf\sim Bin\left( E,PDR \right)$^¥^ |
| Eggs per female (*EFD*) | Brière | 8.56e^-03^ | 14.58 | 34.61 | 𝐸𝑔𝑔𝑠~𝑃𝑜𝑖𝑠𝑠𝑜𝑛(𝐸𝐹𝐷) |
| Mosquito development rate (*MDR*) | Brière | 7.86e^-05^ | 11.36 | 39.17 | 𝑟=𝑀𝐷𝑅∙𝑝𝐸𝐴∙𝑓(𝐷)  𝐸𝑚~𝐵𝑖𝑛(*N_L_*,𝑟)^§^ |
| Egg-to-adult survival probability (*pEA*) | Quadratic | -5.99e^-03^ | 13.56 | 38.29 |  |

* *N_B_*, the number of mosquitoes at a given location biting that specific day.

^+^ *N_D_*, the number of deaths happening in a given day at a specific location.

^†^ Parameters *b* and *c* are the components of vector competence (*vc*).

^‡^ *E*, number of exposed mosquitoes, *N_bit_* refers to those mosquitoes that bit an infected individual, so they are moved to exposed infection status with a probability *vc*.

^¥^ *inf* refers to the moment when a mosquito is moved to infectious stage which is determined by a rate *PDR*

^§^ *Em*, number of emerged mosquitoes, *N_L_* is the number of larvae at a given location.

# References

1. Huber, J.H.; Childs, M.L.; Caldwell, J.M.; Mordecai, E.A. Seasonal temperature variation influences climate suitability for dengue, chikungunya, and Zika transmission. *PLoS Negl Trop Dis* **2018**, *12*, e0006451, doi:10.1371/journal.pntd.0006451.

2. Caldwell, J.M.; LaBeaud, A.D.; Lambin, E.F.; Stewart-Ibarra, A.M.; Ndenga, B.A.; Mutuku, F.M.; Krystosik, A.R.; Ayala, E.B.; Anyamba, A.; Borbor-Cordova, M.J.; et al. Climate predicts geographic and temporal variation in mosquito-borne disease dynamics on two continents. *Nat Commun* **2021**, *12*, 1233, doi:10.1038/s41467-021-21496-7.
