# Supplementary material for: Mobility and non-household environments: Understanding dengue transmission patterns in urban contexts
Source: PLoS Negl Trop Dis. 2026 Jul 2;20(7):e0014487. doi: 10.1371/journal.pntd.0014487 (PMC13354100; doi:10.1371/journal.pntd.0014487)
Supplement: S2 Table — Three levels of human movement were assessed: 100% (base level of movement), 50%, and 20%. Descriptive values for 200 runs include the median of the total number of infections, interquartile range (IQR), and the proportion of infections that occur in any of the five different types of NH environments. (DOCX) [file pntd.0014487.s012.docx]

**S2 Table: Descriptive outcomes for 200 runs at different level of human movement from HH to NH in Kenyan cities of Kisumu and Ukunda**. Three levels of human movement were assessed: 100% (base level of movement), 50%, and 20%. Descriptive values for 200 runs include the median of the total number of infections, interquartile range (IQR), and the proportion of infections that occur in any of the five different types of NH environments.

| **City** | **Human movement’s level** | **Median** | **IQR** | **Proportion of infections in NH** |
| --- | --- | --- | --- | --- |
| Kisumu | 20 | 154 | 108 – 232 | 0.423 |
|  | 50 | 764 | 349 – 1626 | 0.588 |
|  | 100 | 4228 | 3025 – 4921 | 0.670 |
| Ukunda | 20 | 3544 | 1416 – 4716 | 0.531 |
|  | 50 | 8770 | 8305 – 9428 | 0.699 |
|  | 100 | 9416 | 8923 – 10028 | 0.724 |
