# Supplementary material for: Mobility and non-household environments: Understanding dengue transmission patterns in urban contexts
Source: PLoS Negl Trop Dis. 2026 Jul 2;20(7):e0014487. doi: 10.1371/journal.pntd.0014487 (PMC13354100; doi:10.1371/journal.pntd.0014487)
Supplement: S3 Table — Three urban conformations were assessed: Scattered (when non-household (NH) environments is randomly distributed in space), centered (when most of NH are grouped in the center), and clustered (when NH are grouped in three clusters). Descriptive values for 200 runs include the median of the total number of infections, interquartile range (IQR), and the proportion of infections that occur in any of the five different types of NH environments. (DOCX) [file pntd.0014487.s013.docx]

**S3 Table: Descriptive outcomes for 200 runs at different urban conformations of non-household environments for Kenyan cities of Kisumu and Ukunda**. Three urban conformations were assessed: Scattered (when non-household (NH) environments is randomly distributed in space), centered (when most of NH are grouped in the center), and clustered (when NH are grouped in three clusters). Descriptive values for 200 runs include the median of the total number of infections, interquartile range (IQR), and the proportion of infections that occur in any of the five different types of NH environments.

| **City** | **Urban conformation** | **Median** | **IQR** | **Proportion of infections in NH** |
| --- | --- | --- | --- | --- |
| Kisumu | Scattered | 4672 | 3956 – 5227 | 0.678 |
|  | Centered | 4432 | 3587 – 5027 | 0.669 |
|  | Clustered | 3178 | 1785 – 4179 | 0.657 |
| Ukunda | Scattered | 10254 | 9909 – 10596 | 0.754 |
|  | Centered | 9074 | 8744 – 9524 | 0.706 |
|  | Clustered | 9067 | 8784 – 9398 | 0.716 |
