# Supplementary material for: Mobility and non-household environments: Understanding dengue transmission patterns in urban contexts
Source: PLoS Negl Trop Dis. 2026 Jul 2;20(7):e0014487. doi: 10.1371/journal.pntd.0014487 (PMC13354100; doi:10.1371/journal.pntd.0014487)
Supplement: S4 Table — Three levels of intra-urban distance from HH to NH were evaluated: the closest NH location from HH (categorized as zero), at least 500 meters away, and at least 1000 meters away. The table shows the descriptive outcomes for4 200 runs which include the median of the number of cases, the interquartile range (IQR), and the proportion of infections happening in any of the five different types of NH environments. (DOCX) [file pntd.0014487.s014.docx]

**S4 Table: Distance traveled by people from HH to NH have little effect on total burden of dengue.** Three levels of intra-urban distance from HH to NH were evaluated: the closest NH location from HH (categorized as zero), at least 500 meters away, and at least 1000 meters away. The table shows the descriptive outcomes for4 200 runs which include the median of the number of cases, the interquartile range (IQR), and the proportion of infections happening in any of the five different types of NH environments.

| **City** | **Distance traveled from HH to NH (meters)** | **Median** | **IQR** | **Proportion of infections in NH** |
| --- | --- | --- | --- | --- |
| **Kisumu** | 0 (closest) | 3,742 | 2,320 – 4,671 | 0.662 |
|  | 500 | 4,284 | 3,170 – 5,028 | 0.674 |
|  | 1000 | 4,432 | 3,642 – 4,992 | 0.669 |
| **Ukunda** | 0 (closest) | 9,534 | 9,024 – 10,255 | 0.726 |
|  | 500 | 9,408 | 8,958 – 9,942 | 0.724 |
|  | 1000 | 9,269 | 8,785 – 9,965 | 0.721 |
