# Supplementary material for: Mobility and non-household environments: Understanding dengue transmission patterns in urban contexts
Source: PLoS Negl Trop Dis. 2026 Jul 2;20(7):e0014487. doi: 10.1371/journal.pntd.0014487 (PMC13354100; doi:10.1371/journal.pntd.0014487)
Supplement: Attachment — Submitted filename: Response to reviewer.pdf [file pntd.0014487.s018.pdf]

May 15th, 2026

Editorial board
PLOS NEGLECTED TROPICAL DISEASES

Dear editors,

We are pleased to resubmit the revised version of our manuscript entitled "*Mobility and non-household environments: understanding dengue transmission patterns in urban contexts*" for consideration in **PLoS Neglected Tropical Diseases**.

We sincerely thank you and the reviewers for the careful evaluation of our work and for the constructive comments provided. We have carefully revised the manuscript in response to all suggestions and believe that the changes have substantially improved the clarity and robustness of the study.

A detailed, point-by-point response to the reviewers' comments is following provided. As requested, we have submitted both a version of the manuscript with tracked changes and a clean version. In addition to the revisions requested by the reviewers, we have updated the affiliation of one co-author to reflect their current institutional position.

We hope that the revised manuscript is now suitable for publication in **PLoS Neglected Tropical Diseases**, and we remain at your disposal for any further revisions.

Thank you for your time and consideration.

Sincerely,

Víctor Hugo Peña-García
On behalf of all authors

Responses to reviewer comments:

Review of Mobility and non-household environments PNTD

This paper extends the previously published work describing the agent based model designed to examine the extent to which non-household rather than household level infections contribute to overall infection rates using synthetic populations, mosquito oviposition sites and distributions of non-household sites based on "real world" data.

The current paper integrates human mobility, specifically that from households to non-household sites, both in terms of distance travelled and the extent of clustering of the non-household places. This is an interesting and more than incremental advance on the previous paper.

We thank the reviewer for the careful review of our manuscript and the helpful comments, which we have worked to address and incorporate.

I have a few comments and potential suggestions that might improve the paper.

The basic premise that households are considered as the best site to implement vector control is a little odd. I don't know about Kisumu, but in SE Asia, although households are encouraged to employ larvicides, most of vector control is in response to infections and often will include fumigation of entire schools, as children make up the majority of symptomatic infections or whole communities.

It is great that environments other than households are regularly included in vector control activities in SE Asia, an extensive area comprising many endemic settings. Unfortunately, it is not the same in other regions of the world such as Africa and Latin America. In most of these settings, it's very common to follow conventional guidelines like those from WHO (*Vector control operations framework for Zika virus* [1] or *Dengue guidelines for diagnosis, treatment, prevention and control* [2]) where explicit focus on household is made. For Latin America, for instance, the manual from Pan-American Health Organization is explicitly made for indoor spraying (*Manual for indoor residual spraying in urban areas for Aedes aegypti control* [3]).

Control in most of these settings are also guided by entomological indices by recommendation of WHO. Those are estimated considering households as the unit of these indices, such as household (proportion of positive households) and Breteau (number of positive containers in 100 houses) indices. The use of these indices reinforces the idea of households as transmission focus.

To support the idea of higher preference for households as control focus while providing a proper rationale for this assumption, we modified the second paragraph of introduction and added references illustrating the idea, as follows:
"Historically, the main strategy to control the disease has been to reduce vector-human contact by reducing the size of mosquito populations. Although this may not hold in all settings, particularly in parts of Southeast Asia, a long-standing conventional view is that transmission occurs predominantly within households, where susceptible individuals spend much of their time and coexist with biting and breeding vectors. This assumption has driven a substantial body of research to focus on prevention in domestic environments [5–7]. Accordingly, vector control guidelines have largely prioritised interventions in these settings [8–10]."

Another comment is that the current paper requires a very complete knowledge of the previous papers, regarding seroprevalence studies, mosquito breeding sites and the ABM. Whilst giving all details is obviously not necessary, in some places some detail would be helpful. For example, no explanation of the variables and values in equation 2 are given. Also, it states that a prevalence of 0.08% is used, based on a previous seroprevalence study. Is this IgM or IgG? i.e. recent infections or lifetime exposure?

We thank the reviewer for this observation. We revised the manuscript to improve its self-contained readability and reduce the need for familiarity with previous publications. In particular, we added clearer descriptions of model variables and parameters, expanded the explanation of the ABM structure and initialization procedures, and clarified the interpretation and use of empirical data sources such as seroprevalence estimates.

Thirdly, it's not very obvious how age structure in terms of mobility and susceptibility was built in. As the paper is based on a real-life situation in Kisumu, it would be helpful to know what the age-seroprevalence curve is like in Kisumu or at least the average age of first infection. Children will visit a very limited number of non-household sites as compared to adults, likely visit at different times and will also have a very different lifetime exposure to infection.

We thank the reviewer for this important comment. We agree that age influences mobility patterns and that was a central part of the model. However, because we did not have access to age-specific seroprevalence data for the entire population and study time (previous works like the one from Inziani where a baseline seroprevalence was obtained didn't include adults [4], which comprises majority of population), we did not incorporate age-specific baseline immunity into model calibration. Instead, we

structured mobility patterns to be driven by age and focused on the exposure and transmission resulting from these mobility patterns.

While assuming differential levels of immunity according to age as initial state would be more realistic, we believe that the way in which the model was developed allows us to better isolate the patterns of mobility and its derived transmission risk, which is also age-structured and is the actual goal of this work.

To better clarify the importance of age in the mobility patterns and resulting transmission pattern, we modified the second paragraph of Subsection "Model overview" from Methods section as follows:

"*The assignment of each individual as student, worker, or neither depends on their age and the age-specific proportion of individuals in such role according to governmental information [30–32]. Thus, students were as young as 3 and up to 17 years old, workers can be individuals from 15 and up to 64 years old, while some individuals can be both of them or none (e.g. toddlers and retired, which are 65 or older).*"

In addition, we included additional lines in discussion stating some limitations. Among them, we included:
"*Additional limitations should be acknowledged. In particular, the initial immune state of the population was not structured by age. This modelling choice was made to avoid introducing poorly supported assumptions and to better isolate the role of age-dependent mobility in shaping transmission patterns*."

It is also a little unclear how the hourly variation in the mosquito biting rate was taken into account, especially with respect to times of visiting different places. Biting rates tend to peak a little after dawn and a little before dusk.

We thank the reviewer for this helpful observation. In the current model, mosquito biting rate was implemented as a daily temperature-dependent rate, following Mordecai and colleagues [5]. Our temperature data consist of daily mean values, and the biting-rate function provides estimates per mosquito per day rather than hourly. Therefore, incorporating within-day variation in biting activity would require additional assumptions about hourly temperature profiles and biting behaviour that are not supported by the available data.

We agree that *Aedes aegypti* biting activity typically peaks shortly after dawn and before dusk, and that ignoring this pattern may affect the absolute number of infections. We now explicitly acknowledge this as a limitation in the manuscript.

Importantly, the model still links transmission risk to the time individuals spend in each location. Consequently, settings where individuals remain for longer periods are more

likely to overlap with peak biting times, partially capturing the effect of within-day variation. We have clarified this point in the revised manuscript as follows:

"*Another limitation is the absence of within-day variation in mosquito biting activity.* Ae. aegypti *are known to exhibit crepuscular biting patterns, typically peaking shortly after dawn and before dusk, which are not explicitly represented in the model. Biting rate was implemented as a daily temperature-dependent parameter, consistent with the temporal resolution of the available temperature data. Nevertheless, the probability of mosquito–human contact in each setting depends on both the number of individuals present and the duration of their stay. Locations where individuals spend longer periods therefore have a higher likelihood of overlapping with peak biting hours, partially mitigating the impact of not explicitly modelling hourly biting variation.*"

The Results and Discussion sections are very clear and well written.

We thank the reviewer for this positive assessment of the clarity of the Results and Discussion sections.

References cited in this response:

1. World Health Organization. Vector control operations framework for Zika virus. Geneva, Switzerland: World Health Organization; 2016. Report No.: WHO/ZIKV/VC/16.4.

2. World Health Organization, Special Programme for Research and Training in Tropical Diseases, editors. Dengue: guidelines for diagnosis, treatment, prevention, and control. New ed. Geneva: World Health Organization; 2009.

3. Pan American Health Organization. Manual for Indoor Residual Spraying in Urban Areas for Aedes aegypti Control. Washington, D.C.: Pan American Health Organization; 2019. Available: https://iris.paho.org/handle/10665.2/51637

4. Inziani M, Adungo F, Awando J, Kihoro R, Inoue S, Morita K, et al. Seroprevalence of yellow fever, dengue, West Nile and chikungunya viruses in children in Teso South Sub-County, Western Kenya. Int J Infect Dis. 2020;91: 104–110. doi:10.1016/j.ijid.2019.11.004

5. Mordecai EA, Cohen JM, Evans MV, Gudapati P, Johnson LR, Lippi CA, et al. Detecting the impact of temperature on transmission of Zika, dengue, and chikungunya using mechanistic models. PLoS Negl Trop Dis. 2017;11: e0005568. doi:10.1371/journal.pntd.0005568
