# Supplementary material for: Mobility and non-household environments: Understanding dengue transmission patterns in urban contexts
Source: PLoS Negl Trop Dis. 2026 Jul 2;20(7):e0014487. doi: 10.1371/journal.pntd.0014487 (PMC13354100; doi:10.1371/journal.pntd.0014487)
Supplement: Attachment — Submitted filename: Review of Mobility and non.docx [file pntd.0014487.s016.docx]

Review of Mobility and non-household environments PNTD

This paper extends the previously published work describing the agent based model designed to examine the extent to which non-household rather than household level infections contribute to overall infection rates using synthetic populations, mosquito oviposition sites and distributions of non-household sites based on “real world” data.

The current paper integrates human mobility, specifically that from households to non-household sites, both in terms of distance travelled and the extent of clustering of the non-household places. This is an interesting and more than incremental advance on the previous paper.

I have a few comments and potential suggestions that might improve the paper.

The basic premise that households are considered as the best site to implement vector control is a little odd. I don’t know about Kisumu, but in SE Asia, although households are encouraged to employ larvicides, most of vector control is in response to infections and often will include fumigation of entire schools, as children make up the majority of symptomatic infections or whole communities.

Another comment is that the current paper requires a very complete knowledge of the previous papers, regarding seroprevalence studies, mosquito breeding sites and the ABM. Whilst giving all details is obviously not necessary, in some places some detail would be helpful. For example, no explanation of the variables and values in equation 2 are given. Also, it states that a prevalence of 0.08% is used, based on a previous seroprevalence study. Is this IgM or IgG? i.e. recent infections or lifetime exposure?

Thirdly, it’s not very obvious how age structure in terms of mobility and susceptibility was built in. As the paper is based on a real-life situation in Kisumu, it would be helpful to know what the age-seroprevalence curve is like in Kisumu or at least the average age of first infection. Children will visit a very limited number of non-household sites as compared to adults, likely visit at different times and will also have a very different lifetime exposure to infection.

It is also a little unclear how the hourly variation in the mosquito biting rate was taken into account, especially with respect to times of visiting different places. Biting rates tend to peak a little after dawn and a little before dusk.

The Results and Discussion sections are very clear and well written.
